# COVID-related disruptions to colorectal cancer screening, diagnosis, and treatment could increase cancer Burden in Australia and Canada: A modelling study

**DOI:** 10.1371/journal.pone.0296945

**Published:** 2024-04-01

**Authors:** Joachim Worthington, Zhuolu Sun, Rui Fu, Jie-Bin Lew, Kelvin K. W. Chan, Qing Li, Antoine Eskander, Harriet Hui, Kirstie McLoughlin, Michael Caruana, Stuart Peacock, Jean Hai Ein Yong, Karen Canfell, Eleonora Feletto, Talía Malagón

**Affiliations:** 1 The Daffodil Centre, Kings Cross, New South Wales, Australia; 2 Canadian Partnership Against Cancer, Toronto, ON, Canada; 3 ICES, Toronto, Ontario, Canada; 4 Institute of Health Policy, Management and Evaluation, Dalla Lana School of Public Health, University of Toronto, Toronto, Ontario, Canada; 5 Department of Otolaryngology–Head and Neck Surgery, University of Toronto, Toronto, Ontario, Canada; 6 Sunnybrook Health Sciences Centre, University of Toronto, Toronto, Ontario, Canada; 7 Canadian Centre for Applied Research in Cancer Control, Canada; 8 Faculty of Health Sciences, Simon Fraser University, Burnaby, BC, Canada; 9 BC Cancer Canadian Centre for Applied Research in Cancer Control (ARCC), British Columbia, Canada; 10 Department of Oncology, McGill University, Montréal, Canada; 11 St Mary’s Research Centre, Montréal West Island CIUSSS, Montréal, Canada; Lorestan University of Medical Sciences, ISLAMIC REPUBLIC OF IRAN

## Abstract

COVID-19 disrupted cancer control worldwide, impacting preventative screening, diagnoses, and treatment services. This modelling study estimates the impact of disruptions on colorectal cancer cases and deaths in Canada and Australia, informed by data on screening, diagnosis, and treatment procedures. Modelling was used to estimate short- and long-term effects on colorectal cancer incidence and mortality, including ongoing impact of patient backlogs. A hypothetical mitigation strategy was simulated, with diagnostic and treatment capacities increased by 5% from 2022 to address backlogs. Colorectal cancer screening dropped by 40% in Canada and 6.3% in Australia in 2020. Significant decreases to diagnostic and treatment procedures were also observed in Australia and Canada, which were estimated to lead to additional patient wait times. These changes would lead to an estimated increase of 255 colorectal cancer cases and 1,820 colorectal cancer deaths in Canada and 234 cases and 1,186 deaths in Australia over 2020–2030; a 1.9% and 2.4% increase in mortality, respectively, vs a scenario with no screening disruption or diagnostic/treatment delays. Diagnostic and treatment capacity mitigation would avert 789 and 350 deaths in Canada and Australia, respectively. COVID-related disruptions had a significant impact on colorectal cancer screening, diagnostic, and treatment procedures in Canada and Australia. Modelling demonstrates that downstream effects on disease burden could be substantial. However, backlogs can be managed and deaths averted with even small increases to diagnostic and treatment capacity. Careful management of resources can improve patient outcomes after any temporary disruption, and these results can inform targeted approaches early detection of cancers.

## Introduction

Colorectal cancer (CRC) is a significant health burden in high-income settings, and one of the most commonly diagnosed cancer in Australia and Canada [1, 2]. Mortality rates have decreased over the past two decades, believed to be led by improvements in treatment and early detection due to screening [3], However, increasing incidence rates among people at younger ages are a significant cause for concern [4, 5], as it is currently unclear how these increases will propagate as these cohorts age. Early detections through routine screening of asymptomatic individuals along with timely cancer diagnoses and treatment are necessary to manage CRC burden.

The COVID-19 pandemic disrupted health systems worldwide, including provision of cancer screening, diagnosis, and treatment [6–13]. The downstream effects of such disruptions are difficult to determine—in the case of screening disruptions [13], the health impact may not be clear for years or decades to come, and predictive modelling is often used to provide insights into the possible changes [14]. It is also challenging to accurately estimate additional time taken to diagnosis and/or treatment, due to difficulties in defining the comparator of when and which diagnoses and treatments would have occurred in the absence of the pandemic. Reported cancer cases and deaths do not provide a complete or timely picture of the impact of disruptions to screening; in most jurisdictions there are significant delays between data releases [15] and these data are rarely sufficiently granular to identify all delays to treatment. To address these difficulties, predictive epidemiological modelling has been used to estimate the impact of disruptions to screening [16, 17] and hypothetical impacts of disruptions to treatment [3, 18]. Modelling studies to date have simulated hypothetical impacts on screening, diagnosis, and disruption in Australia and Canada [10, 16, 19, 20] and the impact of COVID-19 on delays to diagnosis and treatment in Canada [20]; these studies used hypothetical or incomplete data in the absence of more complete data.

This study aims to provide estimates of the impact of COVID-19 on CRC incidence and mortality incorporating impacts on CRC screening, delays to CRC diagnosis, and delays to CRC treatment in Australia and Canada. The estimates are informed up-to-date real-world data sources and novel scenario modelling is included to provide health systems and policymakers with guidance on resource planning to facilitate long-term CRC burden mitigation.

## Methods

We used a comparative modelling approach, using independent models developed for the Australian and Canadian settings. Both settings used a hybrid modelling approach, combining results from separate microsimulation screening and survival models to assess the combined impacts of screening, diagnostic, and treatment delays on cancer incidence and mortality. Real-world data on screening, diagnoses, and treatment were used as inputs to three models–OncoSim and the McGill cancer model for Canada, and Policy1-Bowel for Australia. These models were used to simulate the relevant populations and estimate CRC incidence and mortality in “pandemic” and “no pandemic” scenarios to estimate the impact of disruptions.

**Fig 1:**
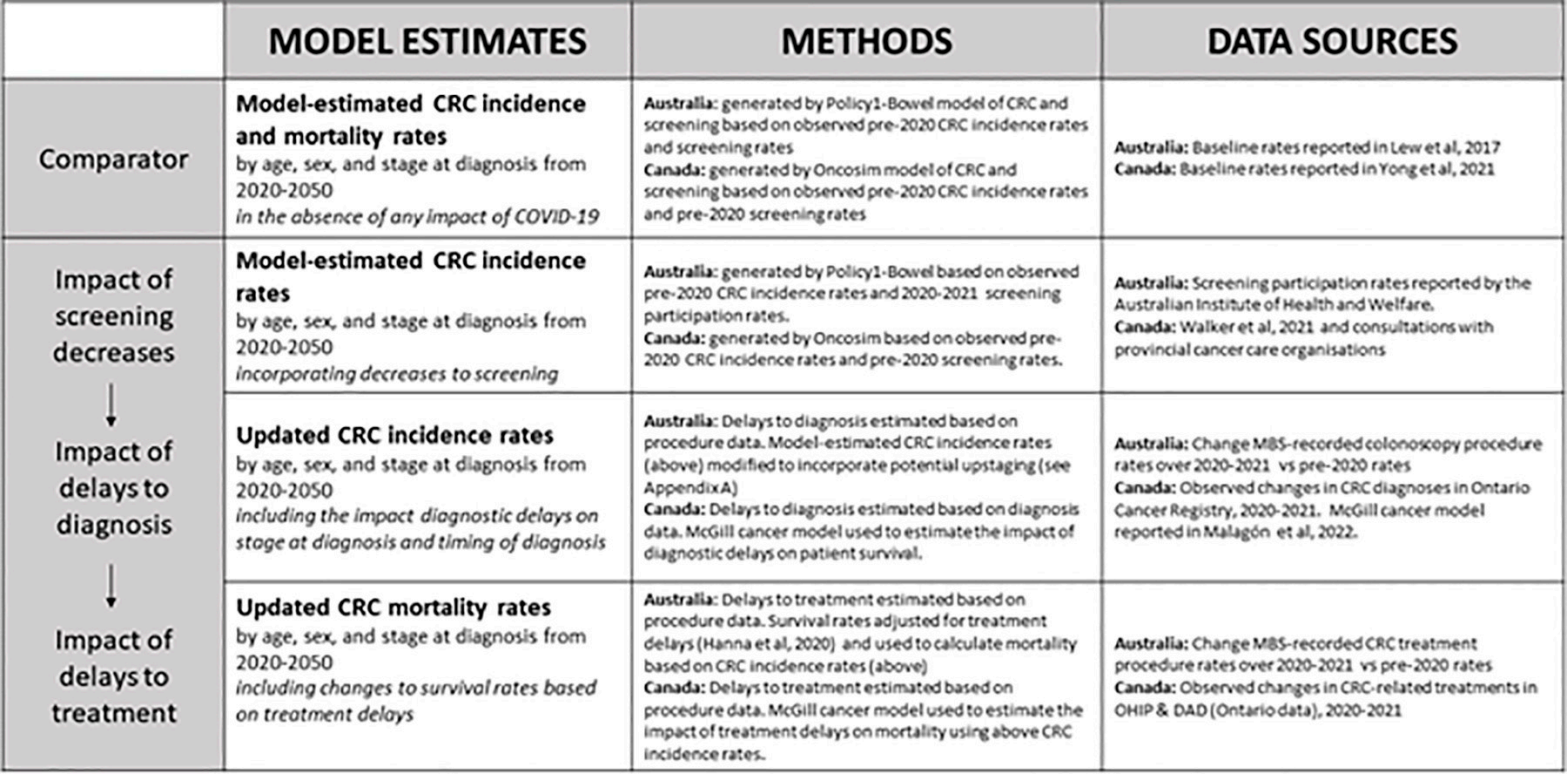
Summary of modelling methods—incorporating real-world data into colorectal cancer burden calculations. MBS–Medicare Benefits Schedule. CRC–colorectal cancer. OHIP–Ontario Health Insurance Plan. DAD–Discharge Abstract Database. Additional methods are included in Appendix A in S1 Appendix.

An overview of the modelling methods is shown in Fig 1. Briefly, estimates for the “no-pandemic” comparator scenario were generated based on observed pre-2020 data. For the pandemic scenarios, there were three stages of modelling–estimating the impact of screening decreases on CRC incidence, estimating delays to CRC diagnosis and the subsequent impact on CRC stage at diagnosis and time of diagnosis, and estimating delays to CRC treatment and the subsequent impact on CRC survival and mortality.

By using a comparative modelling approach, the group was able to compare results generated independently and check the impact of differing methodologies. Data sources and modelling assumptions for each country are detailed below. Full model descriptions and parameters can be found in previous publications [14, 16, 20, 21].

### Screening data

The most up-to-date available data at time of model analysis were identified to inform changes to organised CRC screening over 2020–2021. Pre-2020 screening data was identified as “status quo” rates for the no pandemic comparator and used to calculate changes in screening participation.

In Canada, CRC screening is provided by provincial and territorial programs. Organized CRC screening programs started in 2007 in Canada and are now in place in most provinces, though some opportunistic screening was performed prior to organized screening. While there are differences across provinces, in general screening programs target average-risk individuals between the ages of 50–74 mostly using faecal immunochemical tests (FIT) every 12–24 months as of 2019 [22]. Provincial CRC screening programs vary by the number of samples used for FIT, FIT thresholds, and screening frequency. We used the data on changes in screening volumes during January 2020 to December 2020 reported by Walker et al. for Ontario and used to model the relative change in CRC screening rates for all of Canada in 2020 [23, 24]. Based on the data in this report, and in consultation with other provincial cancer care organisations, we modeled a reduction of 40% in cancer screening in 2020 for Canada. As Walker *et al*. reported that screening volumes had returned to nearly normal levels by December 2020, we assumed screening volumes returned to pre-pandemic levels in 2021 onwards. This was supplemented with consultation with provincial cancer care organisations, who did not report screening volume drops for 2021.

In Australia, screening is provided as biennial FIT by the National Bowel Cancer Screening Program (NBCSP). Screening was first provided in 2006, with a gradual rollout to full screening every two years from age 50–74 from 2019 onwards. We used quarterly screening data from the Australian Institute of Health and Welfare [25] to model screening volume changes from January 2019 to December 2021. Total screening volume was compared to “status quo” 2019 levels to calculate a relative drop in participation. Data on screening invitations were not used in this calculation due to changes and inconsistencies in the way these data were recorded by the National Cancer Screening Register over 2019–2022 [26].

The data sources we used above captured changes in screening test volumes, but not other changes in screening practices. Our analysis therefore only captures impacts of the pandemic on screening participation rates, but not other potential impacts such as follow-up and treatment of screen positives. Any changes to screening outside of organised screening programs in each country was not captured in this study.

### Modelling the impact of changes to screening volume

The impact of the short-term screening participation changes described above on long-term CRC outcomes was estimated by using these decreases in screening participation as inputs for setting-specific microsimulation models: OncoSim in Canada, and Policy1-Bowel in Australia. These natural history models estimate how screening impacts the natural history of CRC in order to estimate changes in CRC incidence, stage at diagnosis, and mortality.

OncoSim is a microsimulation model using Canadian demography data to simulate cancer in the Canadian population. OncoSim-Colorectal, the submodel which simulates CRC (referred to hereafter as OncoSim), assumes that CRCs develop via the conventional adenoma-carcinoma pathway, and adenomas can grow into preclinical cancer or regress (S4 Fig in the provided supplementary materials). OncoSim has been calibrated to adenoma prevalence and Canadian CRC incidence and mortality. Full details of the OncoSim model and how it estimates the impact of CRC screening interruptions during the Covid-19 pandemic have been published previously [16, 21].

OncoSim models the most common screening practice across Canada, which is biennial FIT screening for people aged 50–74 years old. Individuals are simulated from birth to death, either from CRC or other causes. Screening in the model starts in 2008, with a gradual rollout to full screening every two years from age 50–74 for average-risk individuals. In addition, colonoscopy screening in the model starts in 1990 every 5 years from age 40–74 for high-risk individuals with family histories.

*Policy1-Bowel* is a calibrated and validated microsimulation model of CRC and NBCSP screening in Australia [14]. The model simulates the development of pre-cancerous lesions and CRC via two biological pathways (the conventional adenoma-carcinoma pathway and the serrated pathway) in individuals. Individuals are simulated from age 20 to death, either from CRC or other causes. Screening is simulated from 2006, including the gradual rollout to 2019. Only organised screening through the NBCSP was simulated; out-of-program screening was assumed to be unaffected. Full details of *Policy1-Bowel* including model parameters have been published previously [14].

In both settings, the full population was simulated, to assess the population level impacts on CRC outcomes. In Australia, people aged 20–99 in 2020–2021 were simulated to estimate impacts on the affected cohorts; in Canada, OncoSim simulated the full Canadian population born in 1872–2051, accounting for immigration and emigration.

### Data on CRC-related diagnoses and treatment procedures

There are many challenges associated with directly calculating a delay to diagnosis or treatment, particularly when timely data are required–there is a significant lag before the reporting of cancer diagnosis and treatment outcomes, typically of multiple years. Where high-quality data to inform a disruption were not available, proxy measures were used instead. This was completed by estimating the relationship between changes to the observed number of diagnostic or treatment related procedures and additional wait times beyond status quo.

Data on CRC diagnoses and treatments in Canada were obtained for the province of Ontario, which has a single-payer healthcare system, the Ontario Health Insurance Plan (OHIP) [27]. CRC diagnosis records were obtained from the Ontario Cancer Registry (OCR), a population-based registry capturing 96% of index cancers across the province [28, 29]. Receipt of CRC surgery was determined from Canadian Institute for Health information (CIHI)’s Hospital Discharge Abstract Database and Same-Day Surgery database, and confirmed with the diagnosis records from the OCR to ensure the surgical procedure matched with the cancer site and that the procedure was a resection rather than a biopsy [8, 12, 30]. Systemic therapy and radiotherapy visits were determined using physician billing from the OHIP claims database. We restricted systemic therapy to be physician-supervised intravenous infusions (billable using the OHIP G-codes) since oral agents were not robustly captured in the physician billing database. These datasets were linked using unique encoded identifiers and analysed at ICES, an independent, non-profit research institute whose legal status under Ontario’s health information privacy law allows it to collect and analyse health care and demographic data without consent for health system evaluation and improvement. Due to the open nature of these datasets, only data up to October 2021 were used as the data became less reliable after this date due to lags in reporting. Volumes of diagnoses and cancer treatments from March 2020-October 2021 were compared with same month average volumes in 2018–2019 to calculate monthly relative changes in expected cancer-related procedures during the pandemic.

In Australia, CRC diagnostic and treatment procedures were ascertained using Medicare Benefits Schedule (MBS) items using codes identified by Cancer Australia [6, 31] (see Appendix A in S1 Appendix). The identified diagnostic procedures were primarily colonoscopy procedures. A decrease in diagnostic procedures may not directly lead to a proportional decrease in actual diagnoses; for instance, patients at a higher risk may have been more likely to attend diagnostic procedures through the pandemic than patients at lower risk. To address this, changes in diagnostic procedures were compared to changes in cancer notifications in areas where these numbers were available, and this relationship was used to inform adjustments to the inferred decrease in diagnoses.

In Australia, data on actual CRC diagnoses in 2020 were only available for one region, Victoria. Based on these data, the magnitude of the relative decrease in CRC diagnoses in Victoria was 50.4% of magnitude of the relative decrease in colonoscopy procedures in Victoria [32]. In the absence of other data on CRC diagnoses in Australia, any observed change in diagnostic procedures in Australia was assumed to lead to a corresponding decrease in CRC diagnoses with 50.4% magnitude.

### Estimating delays to CRC diagnosis and treatment

Using the data above, we estimated the extent of short-term delays for patients to CRC diagnosis and treatment. The impact of delays to CRC diagnosis and treatment were modelled based on methodology developed previously by Malagón et al. [20] Briefly, it is assumed that observed declines in diagnostic and treatment volumes during the pandemic reflect delays in diagnosis and treatment of cancers that would normally have been diagnosed and normally been treated. These periods where diagnoses and treatments are lower than expected create backlogs of undiagnosed cancers and of untreated patients who experience delays in their diagnosis and treatment.

For Canada, this process is simulated by directly modelling the backlogs using the McGill cancer model, a microsimulation-type agent-based model. Each individual with cancer enters the model in a state of requiring a procedure (diagnosis or treatment), and on a 2-week timestep has a probability of receiving that procedure based on the current procedure capacity and the number of patients currently waiting for that procedure. Expected diagnostic capacity during each time step is based on cancer incidence rates from years prior to 2020 [33]. Expected treatment capacity during each time step is based on usual time to cancer treatment intervals measured prior to the pandemic [34]. From March 2020, procedure capacities are adjusted by multiplying the expected capacity by the observed relative monthly changes in volumes of each procedure during 2020–2021 described above. Diagnostic and treatment backlogs increase during months with relative reductions in volume and decrease during months with relative increases in volume. The time individuals spend on the diagnostic and treatment backlogs represents the additional diagnostic and treatment delays they experience during their cancer care.

For Australia, this process was modelled by computing the current backlog size and treatment capacity on a weekly basis, and from this calculating the average expected wait time for diagnostic or treatment procedures.

### Modelling the impact of CRC diagnosis and treatment delays

Finally, based on the estimated delays to diagnosis and treatment, the subsequent impact on long-term CRC outcomes was estimated. In the Canadian modelling, the McGill cancer model [20] was used to capture the impact of diagnostic delays for non-screen-detected cancers and of treatment delays for both screen-detected and non-screen-detected cancers. The model used the predicted changes in the incidence of screen-detected and non-screen-detected cancers over time predicted by the OncoSim model as inputs, as well as the proportion of cancer cases that are screen-detected by age and stage. These data were used to attribute different diagnostic delays to screen-detected cancers and non-screen-detected cancers, by age and stage. Diagnostic delays for non-screen-detected cancers and the treatment delays for all cancers were transformed into excess cancer mortality rates using a mortality hazard ratio of 1.06 per 4-week delay, based on the meta-analysis by Hanna et al. [35]. The mortality hazard ratio was not applied to diagnostic delays for screen-detected cancers to prevent double counting, as the excess mortality from diagnostic delays for screen-detected cancers were already captured by OncoSim. The impacts of the pandemic on cancer incidence and mortality were summed over both models to estimate the combined impacts of screening, diagnostic, and treatment delays. For further methods, see model documentation version 2.0 [36].

In the Australian modelling, delays to diagnosis were assumed to potentially have an upstaging effect. Based on annual upstage rates of undiagnosed CRC from *Policy1-Bowel* with status-quo wait times [14], the proportion of cancers which diagnosed at a later stage due to a delay was calculated. For delays to treatment, the impact on CRC mortality was calculate by combining CRC survival rates [37] with survival hazard ratios for delays to treatment from Hanna et al. [35]. These updated rates were then used to calculate the expected CRC mortality based on CRC incidence. For further details see Appendix A in S1 Appendix, S1–S3 Figs.

### Modelling scenarios and outputs

To quantify the effect of the pandemic, the following scenarios were modelled:

1. *No pandemic*: a counterfactual comparator which assumed screening rates, diagnostic capacity, and treatment capacity over 2020–2021 were the same as observed pre-pandemic rates
2. *Pandemic scenarios*: for these scenarios we modelled observed changes to screening, diagnostic capacity and treatment over 2020–2021 with the following assumptions regarding health system capacity in 2022 onwards:
  - *No mitigation*: return to status quo (pre 2020) diagnostic and treatment capacity from 2022 onwards
  - *5% mitigation*: a 5% increase to diagnostic and treatment capacity from 2022 to manage patient backlogs in order to return to pre-pandemic diagnostic and treatment intervals

No mitigation was assumed for screening as a new round of biennial screening would have occurred in 2022 for those who missed screening in 2020, making screening catch-up strategies futile post-2022. These scenarios are shown in Table 1.

**Table 1:** Scenarios analysed.

Scenario	Variable	Before 2020	2020–2021	2022 onwards
**No pandemic (comparator)**	Screening	Observed rates (pre-pandemic)^a^	Status quo (pre-pandemic)^b^	Status quo (pre-pandemic)^b^
	Diagnosis & treatment procedure capacity	Observed rates (pre-pandemic)^a^	Status quo (pre-pandemic)^b^	Status quo (pre-pandemic)^b^
**Pandemic (no mitigation)**	Screening	Observed rates (pre-pandemic)^a^	Observed rates (pandemic))^a^	Status quo (pre-pandemic)^b^
	Diagnosis & treatment procedure capacity	Observed rates (pre-pandemic)^a^	Observed rates (pandemic))^a^	Status quo (pre-pandemic)^b^
**5% mitigation**	Screening	Observed rates (pre-pandemic)^a^	Observed rates (pandemic))^a^	Status quo (pre-pandemic)^b^
	Diagnosis & treatment procedure capacity	Observed rates (pre-pandemic)^a^	Observed rates (pandemic)^a^	5% increase^c^

^a^ “Observed rates” refers to rates observed in the relevant time period.

^b^ “Status quo” refers to observed rates/capacity before 2020 (i.e. pre-pandemic).

^c^ Relative increase compared to status quo.

For each scenario, the number of CRC diagnoses and deaths were estimated, as well as the age-standardised rates (ASRs) using the Segi standard world population [38]. An additional “15% mitigation” was simulated as a supplementary analysis.

## Results

**Fig 2:**
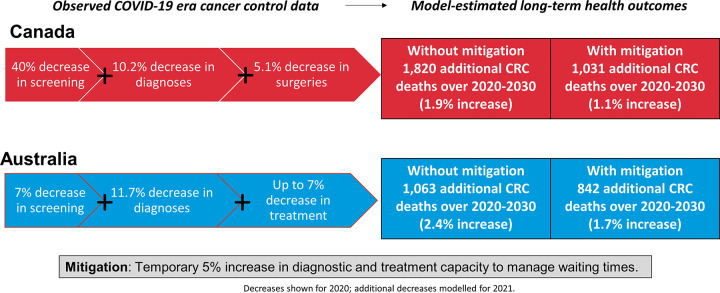
A visual summary of the key study findings.

The key findings are illustrated in Fig 2.

### Observational results and estimates for pandemic impact

Observed and estimated pandemic impacts by country are presented in Table 2. These are derived from the observed data sources as detailed in the methods. In Canada, a 40% drop in screening was estimated in 2020, based on observed data from Ontario; [23] it was estimated that there was no decrease to screening for 2021 as no decreases were recorded. In Australia, a 6.3% drop in screening tests returned was observed in 2020, and a 5.1% drop in 2021.

**Table 2:** Observed changes in resource utilisation and inferred estimated additional waiting time over 2020–2021.

		Screening	Diagnoses		Treatment^a^				
			Procedures / diagnoses^b^	Patients experiencing additional wait^c^	Surgeries	Radiotherapies	Chemotherapies	Overall	Patients experiencing additional wait^c^
**Australia**	2020	-6.3%	-11.7%	16%	-	-	-	+0.1%	1.1%
	2021	-5.1%	-3.4%	29%	-	-	-	-7.2%	24%
**Canada**	2020	-40%	-10.2%	32%	-5.1%	1.5%	3.9%	-	4%
	2021	0%^d^	-0.5%	20%	2.4%	21.5%	14.7%	-	15%

^a^ Treatment was split by type in Canada only—boxes marked “-” were not stratified in the relevant setting. Overall treatment utilization was used in Australia. Yearly total including both increases and decreases to claims over the relevant period on a month-by-month basis.

^b^ Change in diagnostic procedures in Australia (including those with a negative result); change in diagnoses in Canada. Yearly total including both increases and decreases to claims over the relevant period on a month-by-month basis

^c^ Estimated proportion of patients at diagnosis/treatment who waited at least two weeks longer than expected (pre-pandemic) waiting times.

^d^ No decrease in screening was recorded or modelled for Canada in 2021

There was an estimated overall 10.2% decrease in CRC diagnoses for Canada in 2020, and a 0.5% decrease in 2021 based on the observed data from Ontario. Based on modelling, this decline was estimated to lead to 32.2% of patients in 2020 experiencing additional diagnostic waits of at least two weeks, and 19.5% of patients in 2021. In Australia, there was an estimated decrease in CRC diagnoses of 11.7% and 3.4% in 2020 and 2021, respectively, based on observed colonoscopy data. This was expected to lead to 16.1% of patients in 2020 and 28.5% of patients in 2021 experiencing additional waiting times for diagnosis.

In Canada, there were estimated year-on-year increases of 1.5%, and 3.9% to CRC-related radiotherapy and chemotherapy procedures, respectively, in 2020, and a decrease of 5.1% in CRC-related surgeries below expected levels based on data from Ontario. Of note, chemotherapy volumes were observed to increase, not decrease, after March 2020 in Ontario; this increased capacity was reflected in our model estimates. There were short-term declines in CRC-associated radiotherapy volume during the second quarter of 2020; however, these declines were compensated later in 2020 by volume increases, leading to a net positive year-on-year change. The large declines in radiotherapies and surgeries during the Spring of 2020 were accounted for in the calculation of month-on-month delays (see Appendix A in S1 Appendix for monthly volume changes). In 2021 we modelled an increase of 2.4%, 14.7%, and 24.5% in surgery, chemotherapy, and radiotherapy volume, respectively. This was estimated to lead to 3.7% of patients experiencing additional treatment-related delays in 2020, and 14.6% in 2021.

No change in treatment procedures was estimated for 2020 in Australia, as no decreases were reported across all treatment types recorded; in 2021 a 7.2% decrease in any CRC treatment procedures was estimated compared to expected numbers. It was estimated that 1.1% of patients would experience a wait for treatment in 2020 of two or more weeks longer than typical waiting times; in 2021 this would increase to 23.9%.

### Predicted model outcomes

**Fig 3:**
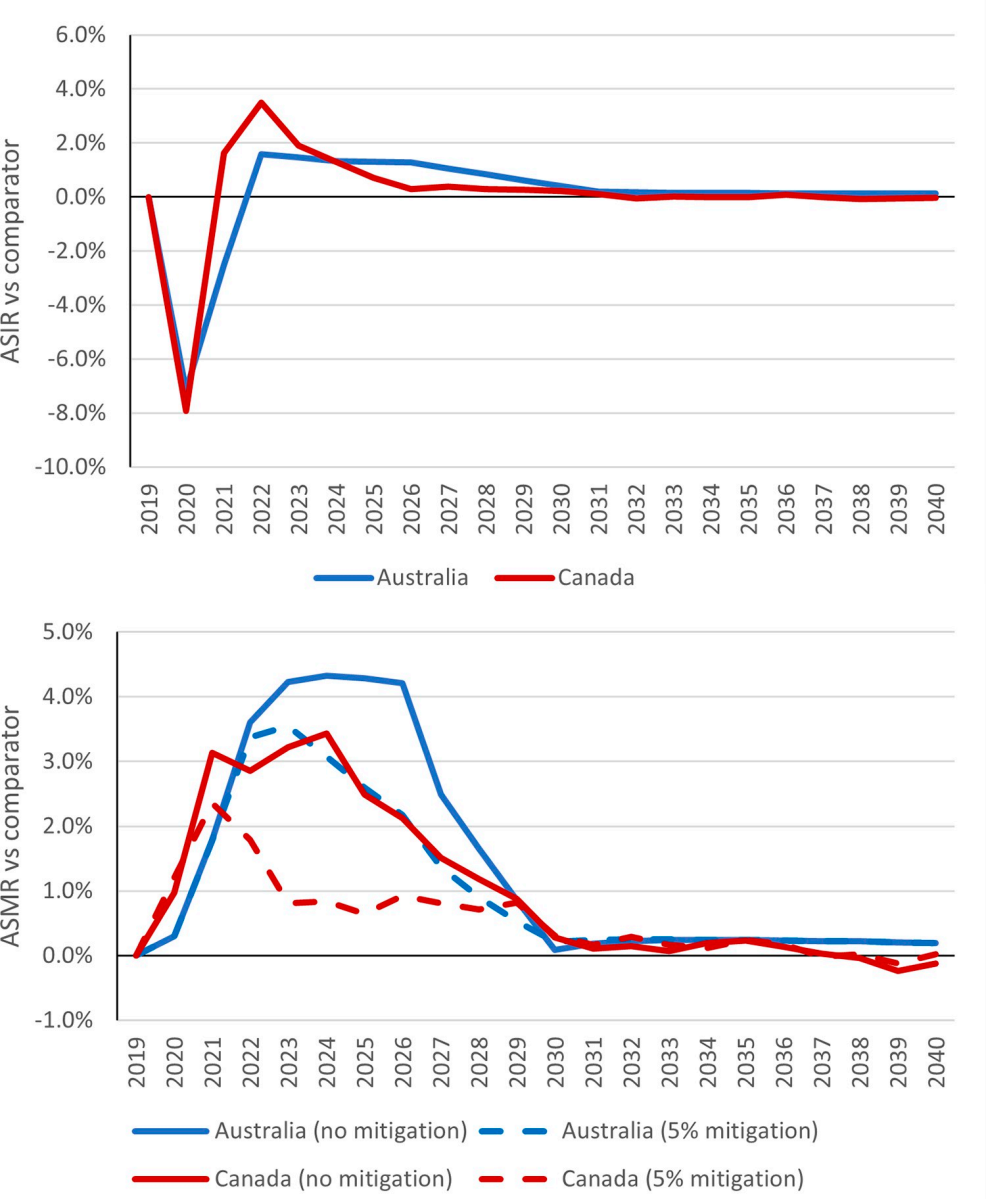
Relative changes in colorectal cancer age-standardized incidence rates (ASIR) (top) and age-standardized mortality rates (ASMR) (bottom) in pandemic scenarios vs no pandemic scenario. The no mitigation scenario represents an assumed return to status quo cancer care volumes in 2022 onwards, while the 5% mitigation scenario represents an assumed 5% increase in treatment capacity from 2022 onwards to resolve accumulated backlogs.

**Fig 4:**
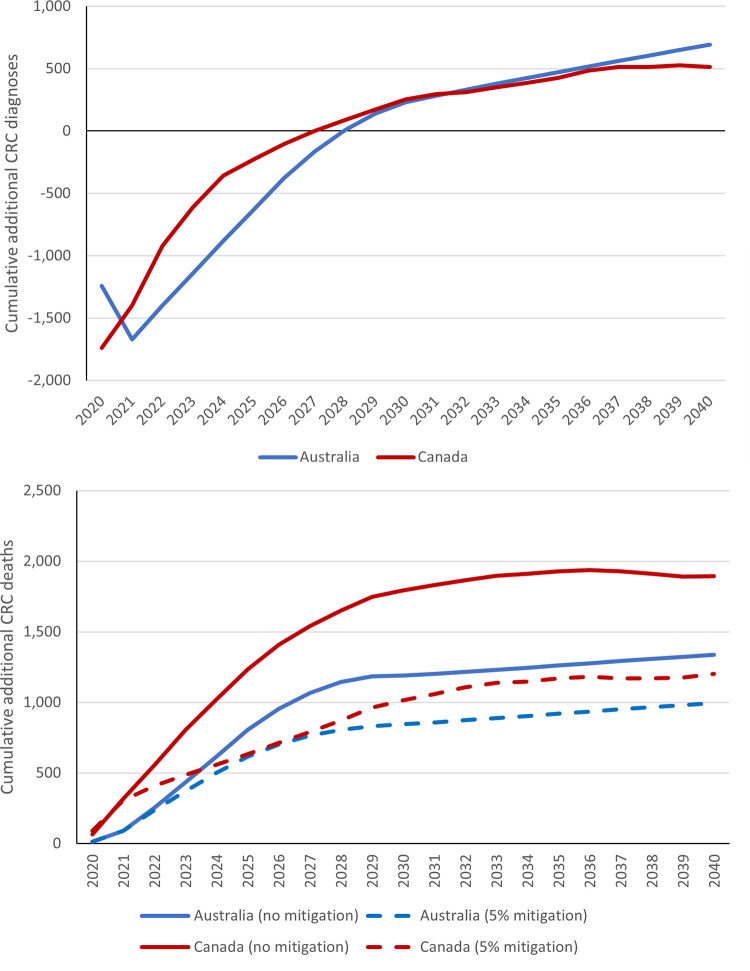
Cumulative additional colorectal cancer (CRC) diagnoses (top) and deaths (bottom) in pandemic scenarios vs no pandemic scenario. The no mitigation scenario represents an assumed return to status quo cancer care volumes in 2022 onwards, while the 5% mitigation scenario represents an assumed 5% increase in treatment capacity from 2022 onwards to resolve accumulated backlogs.

The above estimated changes to screening and additional diagnostic waiting times were used to estimate predicted short-term decreases in CRC incidence, with 1,740 and 1,242 fewer CRC diagnoses estimated in Canada and Australia respectively in 2020 vs the no pandemic comparator (Table 3, Figs 3 and 4). However, our modelling predicts that the decreases in screening are expected to lead to long-term increases in CRC incidence due to missed precancerous lesions developing into CRC. Over 2020–2030, 255 and 234 estimated additional CRC cases would be diagnosed in Canada and Australia respectively due to missed screening opportunities, representing a 0.1% increase in CRC incidence in both settings.

**Table 3:** Estimated short- and long-term cumulative changes in CRC incidence and CRC mortality attributable to screening, diagnosis, and treatment decreases in 2020 and 2021. All results are vs “no pandemic” scenario (see Table 1). Numbers in brackets are relative changes. Includes short-term decreases to incidence due to reductions in diagnoses, and long-term increases due to CRC which were not removed at screening at a precancerous stage.

			2020–2021	2020–2025	2020–2030	2020–2050
**Australia**	**Changes in CRC incidence vs no pandemic**		-1,672 (-4.7%)	-630 (-.6%)	234 (.1%)	1,065 (.1%)
	**Changes in CRC mortality vs no pandemic**	**Screening-related**	-9	40	123	396
		**Diagnoses/treatment related**	100	764	1,063	1,068
		**Total (no mitigation)**	91 (1.1%)	805 (3.1%)	1,186 (2.4%)	1,464 (0.9%)
		**Total (5% mitigation)**	91 (1.1%)	631 (2.4%)	842 (1.7%)	1,114 (0.7%)
**Canada**	**Changes in CRC incidence vs no pandemic**		-1,398 (-3.1%)	-195 (-.2%)	255 (.1%)	443 (.%)
	**Changes in CRC mortality vs no pandemic**	**Screening-related**	-16	194	525	805
		**Diagnoses/treatment related**	334	1,079	1,296	1,070
		**Total (no mitigation)**	318 (2.0%)	1,272 (2.6%)	1,820 (1.9%)	1,875 (.6%)
		**Total (5% mitigation)**	306 (1.9%)	629 (1.3%)	1,031 (1.1%)	1,053 (.3%)

Over the period 2020–2030, the combined impact of disruptions to screening, diagnosis, and treatment were predicted to lead to a 1.9% and 2.4% increase in CRC mortality in Canada and Australia, respectively (Table 3, Figs 3 and 4). This would translate to 1,820 and 1,186 additional deaths respectively. Of these, an estimated 28.8% would be attributable to screening disruptions in Canada, and 10.3% in Australia. If a 5% increase to was made to both diagnostic and treatment capacity from 2022 onwards, this would significantly reduce the CRC mortality increase over 2020–2030 to 1.1% in Canada and 1.7% in Australia.

The results of the 15% increase in capacity are shown in Appendix B in S1 Appendix. A 15% mitigation scenario would reduce the CRC mortality increase over 2020–2030 by 43% in Australia and 58% in Canada, compared to a 29% and 43% reduction in the 5% mitigation scenario.

Over the period 2031–2050, the ongoing impact would primarily be due to the long-term impact of screening decreases (Table 3). This is due to precancerous lesions that would otherwise be caught at screening developing into CRCs.

Additional unprocessed model outputs are included as supplementary material in S1 Data.

## Discussion

This is the first study to estimate the cumulative impact of COVID-19 disruptions to combined CRC screening, diagnosis, and treatment on health outcomes. By incorporating real-world data and including a comparative analysis of two settings from independent modelling teams, this work provides a robust overview of both the burden of disease and the effectiveness of potential mitigation strategies. This modelling can help inform decision making now and during future disruptions, including those unrelated to the pandemic. The results here can guide and focus investment in diagnostic and treatment services to improve resilience in cancer control systems, amid ongoing uncertainty about the true extendt of the impact of COVID-19 on cancer diagnoses [39].

The impact of disruptions to CRC screening were estimated to lead to a short-term reduction in CRC incidence through a downturn in diagnoses, and long-term increases in both CRC incidence and mortality. Delays to diagnosis and treatment are expected to lead to increases in CRC mortality, with the peak increase occurring over 2022–2024 in Canada and 2024–2026 in Australia. Including 5% increases in diagnostic and treatment capacity to deal with patient backlogs would mitigate this increase in mortality significantly. Further increases to capacity had diminishing impacts on long-term CRC benefits, indicating that relatively small increases to capacity may be sufficient to deal with backlogs. By simulating two different high-income settings with independent modelling teams, the results can be compared, including the impacts of modelling assumptions.

The impact of COVID-19 on health systems have been complex and varied [11, 40], and in some settings health systems took a long time to recover. The majority of the recorded impact correlated with waves of COVID cases [41]. Although initial waves of the COVID-19 pandemic had lower case numbers in Australia than other countries [42], strict lockdowns and procedures still impacted access to care [43–45]. It should also be noted that in both Australia and Canada, lockdowns and other restrictions were primarily managed on a state-by-state/province-by-province basis; for instance, Victoria experienced significantly longer formal lockdown periods than other areas of Australia.

Also of note, while Canada had a significantly larger impact of the COVID-19 pandemic and associated mortality than Australia [46], this did not translate into significantly larger predicted pandemic-related CRC care delays and additional cancer mortality. Our results suggest that the crisis responses of health care systems and changes in individuals’ healthcare seeking behaviours during the public health crises are more significant determinants of the negative externalities on other health outcomes than COVID-19 transmission patterns. There are many possible explanations for this, including real or perceived risks of COVID-19 transmission, changes to hospital procedures, and restrictions around freedom of movement; it is unknown which of these impacted health seeking behaviours and by how much.

While direct impacts on health system resources can often be measured directly, the effect of these changes to resources on cancer cases and deaths is indirect and often unclear. There have already been significant impacts on CRC incidence and mortality that may be attributable to COVID-19 in some areas, though the factors underlying these impacts are complex and uncertain. For example, in Victoria, Australia CRC incidence in 2020 and 2011 were more than 10% lower than expected, and CRC mortality rates decreased over 5% [47]. However, these will also be influence by screening and trends in risk factors [3, 48]. Modelling studies like this one can estimate potential consequences from a specific source that may otherwise be unclear.

As with any long-term modelling analysis, there are natural limitations and uncertainty. A key focus of this project was estimating the additional waiting time to diagnosis and treatment attributable to the COVID-19 pandemic. By the nature of this measure, it is impossible to have a complete understanding of the counterfactual—how long a person would have waited for diagnosis and treatment had there not been a pandemic. Instead, these were estimated indirectly based on changes in volumes of relevant procedures or diagnoses. In the absence of detailed data on which cohorts were affected, all delays were assumed to affect all patients equally, regardless of age, sex, or cancer stage. The analysis also could not capture details of changes to treatment, such as patients who changed treatment patterns due to the pandemic. Instead, this was reflected indirectly as a delay to treatment. We also did not directly address the possibility of catch-up screening, i.e. individuals who were assumed to miss screening in 2020/2021 due to the observed decreases would not return to screening until their next invitation round in 2022/2023. In some jurisdictions, individuals who missed screening were encouraged to participate again as soon as possible, regardless of their invitation round. As there were no data to directly estimate this, it was excluded from this study. Previous studies have estimated the hypothetical impact of catch-up screening after COVID-related disruptions [17].

Another limitation of the data is the use of procedures as a proxy of diagnoses for Australia. In Australia, up-to-date data on CRC diagnoses in 2020–2021 was only available in Victoria; as noted above, Victoria had significantly stricter lockdown arrangements than other Australian states over this period. Additionally, the MBS codes used for Australian modelling do not capture all diagnostic and treatment procedures. This is a significant limitation of the data; to address this, our modelling assumed that any drop in non-MBS recorded procedures was proportional to the (known) drop in MBS-recorded procedures, as in previous studies [49].

The differences in modelling approaches for the two settings meant that not all factors could be captured in the same ways. For example, the impact of screening in Australia is also impacted by assumptions around screening participation patterns in individuals who have previously screened. The simulation of diagnostic and treatment is more detailed in the Canadian modelling than the Australian modelling, including breakdowns of treatment types; it is likely to give a more nuanced estimate of the range of delays to services, vs the Australian modelling which assumed a consistent delay across the whole cohort. While the numbers of cases predicted by both models vary by setting, the results across the two modelled settings are nonetheless qualitatively similar and robust. However, the impact of COVID-19 on factors such as CRC screening varied immensely between countries [17]. While the number of excess cancer cases and deaths will therefore vary across countries, the qualitative observation that short-term increases to health system capacity can help mitigate the impact of a disruption is likely to be true in general. This should also be taken in the context of other trends in CRC cases and deaths, including aging populations, changes in risk factors, and improvements in treatment. These factors are likely to outweigh any lasting changes attributable to COVID-19.

There is also a great deal of uncertainty on the true effect of delays on cancer mortality. While we used the estimate based on best available evidence, there is a great deal of both uncertainty and potential for bias in these data, as individual patients may experience delays for clinical reasons. In this modelling, one of the core assumptions is that all cancers are eventually diagnosed due to symptoms and/or death attributed to cancer. However, in reality it is possible that some cancers are overdiagnosed due to screening or incidental findings, and so some of the cancers missed during the pandemic may never be diagnosed.

The methodology for estimating delays in Australia meant that Australian patients were all assumed to experience an average delay to treatment, and outliers who experienced no delay or an exceptionally long delay were not captured, nor were patients who did not receive any treatment at all due to the pandemic. The impact of treatment delays on mortality was based on hazard ratios reported in a review–applying these to a different setting than the original studies may limit their reliability.

A further limitation is that, although the modelling in this study captured the impact of additional waiting times to diagnosis and treatment, this approach could not capture any potential health benefits on shorter waiting times vs “status quo”. Individuals experiencing shorter waits than usual were assumed to have baseline CRC stage at diagnosis and survival rates. For example, in Canada there is evidence that the CRC patients who were already diagnosed and waiting for treatment during the early months of the pandemic experienced shorter times to surgery [9, 34]. This is presumably due to shorter surgical waitlists caused by decreases in cancer diagnoses as well as prioritization of urgent cancer-related surgeries during this time. However, this apparent short-term improvement in wait times does not account for the delays experienced by patients who went undiagnosed and therefore were not on the surgical waitlist, or those who were diagnosed but experienced delays in getting on the surgical waitlist. By basing our assumptions of delays on treatment volumes rather than wait time data, we avoid the inherent selection biases that exists in the calculation of wait times in order to also capture the delays experienced by cancer cases who are yet undiagnosed or experiencing delays before getting on treatment waitlists. This is likely why we estimated higher mortality impacts for Canada using this approach than those from a previous model by Parmar *et al*., [50] who focused their analysis on excess mortality caused by treatment delays experienced by patients already on the surgical waitlist. Our study, along with further evidence, could be used to revise clinical guidelines to ensure more efficient and appropriate use of colonoscopy to reduce wait times.

Delays to screening, diagnosis, and treatment may cause significant anxiety in patients and represent a significant psychological burden. There are also likely unforeseen downstream effects of these disruptions, particularly in screening. Studies have shown that recent screening participation is a strong indicator of future screening behaviour, regardless of previous screening behaviour [51]. This means individuals who miss screening due to the pandemic may be less likely to return to screening, leading to ongoing decreases in participation. Ongoing monitoring is key to ensuring high participation rates, and efforts such as targeted mass media campaigns can be deployed where needed to increase engagement [52, 53].

The COVID-19 pandemic has caused significant difficulties in the continuation of health services, and delays to CRC care is just one example among many. However, we found that even small increases to services can help to manage patient backlogs and mitigate long-term impacts of the pandemic on cancer mortality. Targeted investment and temporary increases to services is necessary to ensure health system resilience and reducing cancer burden, both now and for future disruptions.

## Supporting information

S1 Appendix Additional appendices with further methodology and supplementary results. (DOCX)

S1 Data Model outputs used to inform this study. (XLSX)

S1 Fig Model of CRC stage at diagnosis with delays to diagnosis (Australia). (PNG)

S2 Fig Post- diagnosis CRC survival model (Australia). (PNG)

S3 Fig Modelled monthly changes in colorectal cancer procedure capacity assumed for Canada, based on data from Ontario (treatments and diagnoses) and from OncoSim (diagnoses of screen-detected cancers). See main text for data sources.
(PNG)

S4 Fig Natural history of colorectal cancer, as simulated by Policy1-Bowel and OncoSim. (PNG)
