# Supplementary material for: COVID-related disruptions to colorectal cancer screening, diagnosis, and treatment could increase cancer Burden in Australia and Canada: A modelling study
Source: PLoS One. 2024 Apr 1;19(4):e0296945. doi: 10.1371/journal.pone.0296945 (PMC10984523; doi:10.1371/journal.pone.0296945)
Supplement: S1 Appendix — (DOCX) [file pone.0296945.s001.docx]

# S1 Supplementary Material: COVID-Related Disruptions to Colorectal Cancer Screening, Diagnosis, and Treatment could Increase Cancer Burden in Australia and Canada: a modelling study

## Appendix A – Additional Methods

#### MBS item codes used in Australia

To capture the impact of the COVID-19 pandemic on diagnosis and treatment, data from relevant MBS codes were identified. These codes were identified based on a report by Cancer Australia on early pandemic impact.^1^

The following MBS item codes were used:

- **Diagnostic procedures:** 32072, 32075, 32084, 32087, 32096, 32222, 32223, 32224, 32225,
- 32226, 32227, 32228, 32088, 32089, 32090, 32093
- **Treatment procedures:** 32000, 32003, 32004, 32005, 32006, 32009, 32012, 32024, 32025, 32026, 32028, 32039, 32042, 32045, 32046, 32099, 32102, 32103, 32104, 32105, 32106, 32108, 32015, 32018, 32021, 32023, 32030, 32047, 32051, 32054, 32057

It should be noted that some colonoscopies are not recorded through MBS, and complete data is not available for these.^2^ The modelling assumption used in this study is that the same proportion of non-MBS colonoscopies are used for diagnostic purposes, and that non-MBS colonoscopies were affected in similar ways to MBS colonoscopies.

Although patients are likely to have more than one diagnostic or treatment procedure, the key assumption of this study is that a decrease in procedures corresponds to a proportional decrease in patients being diagnosed and/or undergoing treatment.

#### Modelling methods for Australia

To capture the effects of changes to screening in Australia, and provide estimates of CRC incidence rates by year, age, sex, and stage at diagnosis, the *Policy1-Bowel* model was used. *Policy1-Bowel* is a calibrated and validated model of CRC and screening in Australia. The model simulates the development of precancerous lesions, including conventional and sessile serrate adenomas, on an individual level, to capture the full impact of screening. Parameters for this model can be found in the appendix of Lew et al.^3^

To allow for flexible and modifiable stage at diagnosis and CRC survival, two additional models were developed to supplement the full *Policy1-Bowel* model. These Markov models allowed post-hoc calculation of CRC upstaging and survival based on the CRC incidence rates generated by *Policy1-Bowel.*

The first of these models calculates modified stage at diagnosis, including delays to diagnosis. Using stage-specific CRC incidence rates from *Policy1-Bowel* as a baseline, new stage-specific incidence rates are calculated based on the idea that a delay can induce upstaging from Stage N to Stage N+1 (note that there is no upstaging by multiple stages; the delays modelled are sufficiently short that the likelihood of this occurring is negligible). This is illustrated in Supplementary Figure 1. The probability that a cancer is diagnosed at a later stage is based on the length of the delay before diagnosis. Upstage rates were calculated from *Policy1-Bowel*; as these upstage rates are annual probabilities, these were converted to constant hazard rates which were used to calculate upstage probabilities for relevant delay length. These data are shown in Supplementary Table 1.

To supplement this, a survival model for patients diagnosed with CRC was developed. In this model, every year after diagnosis an individual can die (either from CRC or other causes) or remain a CRC patient, until five-years post diagnosis where they become a CRC survivor. This is illustrated in Supplementary Figure 2. Annual CRC survival rates vary depending on age, sex, CRC stage at diagnosis, and time since CRC diagnosis. In the case of any delay, these rates are further modified based on the likelihood of receiving CRC treatment^4^ and survival hazard ratios for delays to diagnosis.^5^ Other-cause survival was calculated based on lifetables from the Australian Bureau of Statistics.^6^ Survival probabilities are shown in Supplementary Table 2.

#### Additional modelling methods for Canada

Detailed technical documentation on the McGill survival model is provided elsewhere.^7^ Version 2.0 of the model was used for the current study. Version 2.0 of the model includes age-specific time trends since 2000 to predict future colorectal cancer incidence, and structural modifications to allow using predictions from Oncosim as model inputs.

We used the following OHIP fee codes to identify chemotherapy and radiotherapy visits:

- **Chemotherapy:** G339, G345, G359, G381, G281, G382, G388
- **Radiotherapy:** X310, X311, X312, X313, X302, X304, X305, X306, X322, X323, X334, X324, X325, X326, X327, X335, X328, X329, X332, X336, X330

A more detailed breakdown of the monthly changes in CRC-related procedures for Canada is provided in Supplementary Figure 4. See main text for data sources and references used to inform estimates. For screen-detected cancer diagnoses, only yearly data was available from OncoSim; declines in diagnoses were smoothed over the year of 2020 so that the largest declines occurred in April-May 2020. For chemotherapies, the data did not suggest any significant declines had occurred in Spring 2020 across several major cancer sites, so only yearly cumulative estimates of relative changes were calculated for several cancer sites combined (colorectal, breast, cervical, lung) and applied to all months of 2020 and 2021; a 10% permanent increase in chemotherapy capacity was assumed for 2022 onwards in all scenarios given the sustained increase in chemotherapy volumes observed in 2020-2022. Relative capacity for CRC-specific radiotherapy and surgery was assumed to return to 0% change relative to status quo in 2022 for the ‘no mitigation’ scenarios, and to +5% in the ‘5% mitigation’ scenario.

Supplementary Table 1 - Rates used to calculate likelihood of upstaging after a delay to diagnosis in Australia.^3^

| CRC Stage | Annual Upstage Probability^[[1]](#footnote-2)^ | Constant hazard rate for upstaging | 4-week upstage probability^5^ |
| --- | --- | --- | --- |
| Stage 1 | 30% | 0.357 | 2.70% |
| Stage 2 | 60% | 0.916 | 6.79% |
| Stage 3 | 20% | 0.223 | 1.70% |

Supplementary Table 2- Annual cause-specific survival probability for diagnosed patients in Australia, stratified by cancer stage at diagnosis and years since diagnosis.^8^

| Annual cause-specific CRC survival probability | | | |
| --- | --- | --- | --- |
| Stage at diagnosis | Years since diagnosis | | |
|  | 1 | 2-3 | 4-5 |
| Stage 1 | 99.3% | 99.7% | 99.9% |
| Stage 2 | 96.4% | 98.2% | 97.7% |
| Stage 3 | 93.5% | 92.0% | 94.9% |
| Stage 4 | 49.3% | 68.0% | 76.7% |

Supplementary Figure 1 - Model of CRC stage at diagnosis with delays to diagnosis (Australia)


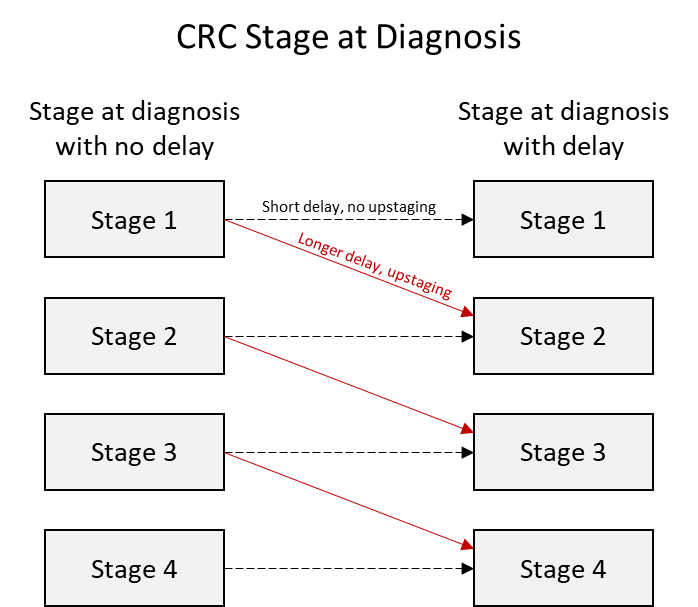


Supplementary Figure 2 - Post- diagnosis CRC survival model (Australia)


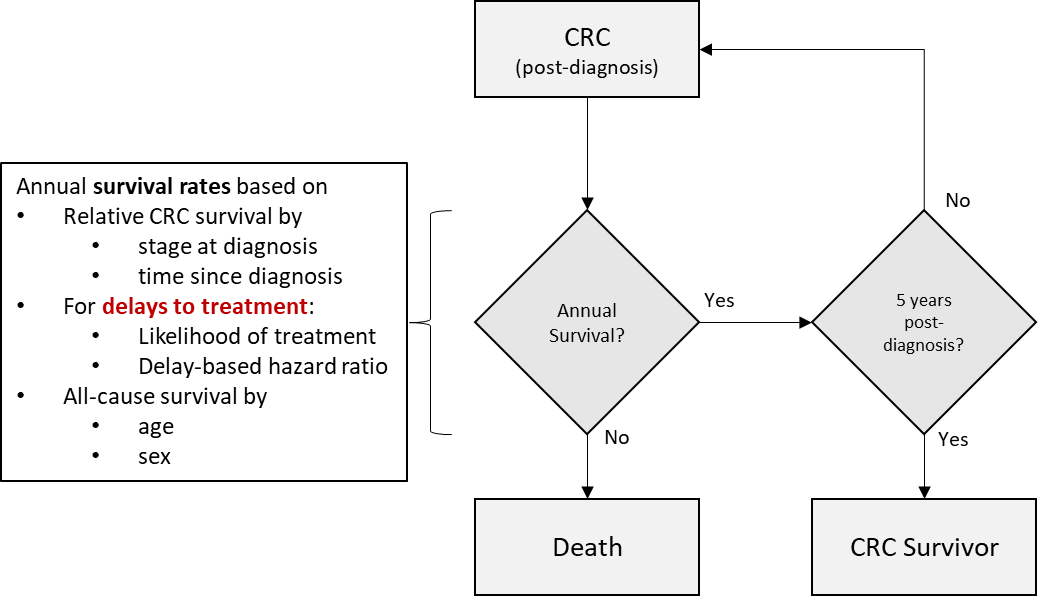


Supplementary Figure 3 – Modelled monthly changes in colorectal cancer procedure capacity assumed for Canada, based on data from Ontario (treatments and diagnoses) and from OncoSim (diagnoses of screen-detected cancers). See main text for data sources.

Supplementary Figure 4

*Natural history of Colorectal Cancer, as simulated by Policy1-Bowel and OncoSim.*

*
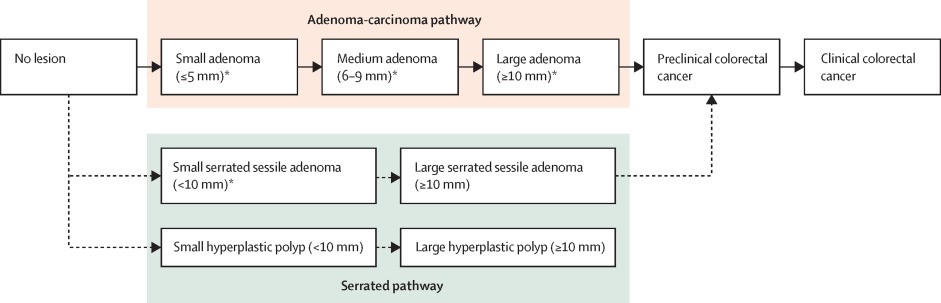
*

Note: only the adenoma-carcinoma pathway is simulated with OncoSim, while both the adenoma-carcinoma pathway and the serrated pathway are simulated with Policy1-Bowel. Policy1-Bowel assumes that small and medium adenomas with high-grade dysplasia and/or villous structure and small hyperplastic polyps can progress to preclinical colorectal cancer.

## Appendix B – Supplementary results

#### 15% mitigation scenario

As an exploratory analysis, an “15% mitigation” scenario was simulated, which assumed a 15% increase to diagnosis and treatment procedure capacity from 2022 onwards (compare to the “5% mitigation” scenario in Table 1). The results of this analysis are shown in Table B.1, compared to the “no mitigation” and “5% mitigation” scenarios.

This analysis found that 5% mitigation would reduce additional CRC deaths over 2020-2030 by 29% in Australia and 43% in Canada, while 15% mitigation would reduce additional CRC deaths by 43% in Australia and 58% in Canada. Considering the increase in resources required, this represents a significantly diminished return on investment.

*Table B.1 - Changes in CRC mortality vs no pandemic, by level of mitigation.*

|  |  | **2020-2021** | **2020-2025** | **2020-2030** | **2020-2050** |
| --- | --- | --- | --- | --- | --- |
| **Australia** | **No Mitigation** | 91 (1.1%) | 805 (3.1%) | 1,186 (2.4%) | 1,464 (.9%) |
|  | **5% mitigation** | 91 (1.1%) | 631 (2.4%) | 842 (1.7%) | 1,114 (0.7%) |
|  | **15% mitigation** | 91 (1.1%) | 499 (1.9%) | 674 (1.4%) | 946 (0.6%) |
| **Canada** | **No mitigation** | 318 (2.0%) | 1,272 (2.6%) | 1,820 (1.9%) | 1,875 (0.6%) |
|  | **5% mitigation** | 306 (1.9%) | 629 (1.3%) | 1,031 (1.1%) | 1,053 (0.3%) |
|  | **15% mitigation** | 288 (1.8%) | 567 (1.1%) | 764 (0.8%) | 897 (0.3%) |

## Bibliography

1. Cancer Australia. The Impact of COVID-19 on Cancer-Related Medical Services and Procedures in Australia in 2020: Examination of MBS Claims Data for 2020, Nationally and by Jurisdiction.; 2021. https://www.canceraustralia.gov.au/the-impact-of-COVID-19-on-cancer-related-medical-services-and-procedures-in-Australia-in-2020

2. Worthington J, He E, Lew JB, St John J, Horn C, Grogan P, Canfell K, Feletto E. Colonoscopies in Australia - how much does the National Bowel Cancer Screening Program contribute to colonoscopy use? Public Health Res Pract. Published online December 8, 2022:32342216. doi:10.17061/phrp32232216

3. Lew JB, St John DJB, Xu XM, Greuter MJE, Caruana M, Cenin DR, He E, Saville M, Grogan P, Coupé VMH, Canfell K. Long-term evaluation of benefits, harms, and cost-effectiveness of the National Bowel Cancer Screening Program in Australia: a modelling study. Lancet Public Health. 2017;2(7):e331-e340. doi:10.1016/S2468-2667(17)30105-6

4. Yap S, He E, Egger S, Goldsbury DE, Lew JB, Ngo PJ, Worthington J, Rillstone H, Zalcberg JR, Cuff J, Ward RL, Canfell K, Feletto E, Steinberg J. Colon and rectal cancer treatment patterns and their associations with clinical, sociodemographic and lifestyle characteristics: analysis of the Australian 45 and Up Study cohort. BMC Cancer. 2023;23(1):60. doi:10.1186/s12885-023-10528-8

5. Hanna TP, King WD, Thibodeau S, Jalink M, Paulin GA, Harvey-Jones E, O’Sullivan DE, Booth CM, Sullivan R, Aggarwal A. Mortality due to cancer treatment delay: systematic review and meta-analysis. BMJ. Published online November 4, 2020:m4087. doi:10.1136/bmj.m4087

6. Australian Bureau of Statistics. Life Tables.; 2022. Accessed January 16, 2023. https://www.abs.gov.au/statistics/people/population/life-tables/latest-release

7. Malagón T. Canada Cancer Incidence, Treatment, and Survival Model Technical Documentation. Published online 2021. doi:10.5683/SP2/REMSZ6

8. Australian Government National Cancer Control Indicators. Relative Survival by Stage at Diagnosis (Colorectal Cancer).; 2019. Accessed January 16, 2023. https://ncci.canceraustralia.gov.au/outcomes/relative-survival-rate/relative-survival-stage-diagnosis-colorectal-cancer

1. In the absence of diagnosis. [↑](#footnote-ref-2)
