# Supplementary figures and images for: COVID-related disruptions to colorectal cancer screening, diagnosis, and treatment could increase cancer Burden in Australia and Canada: A modelling study
Source: PLoS One. 2024 Apr 1;19(4):e0296945. doi: 10.1371/journal.pone.0296945 (PMC10984523; doi:10.1371/journal.pone.0296945)

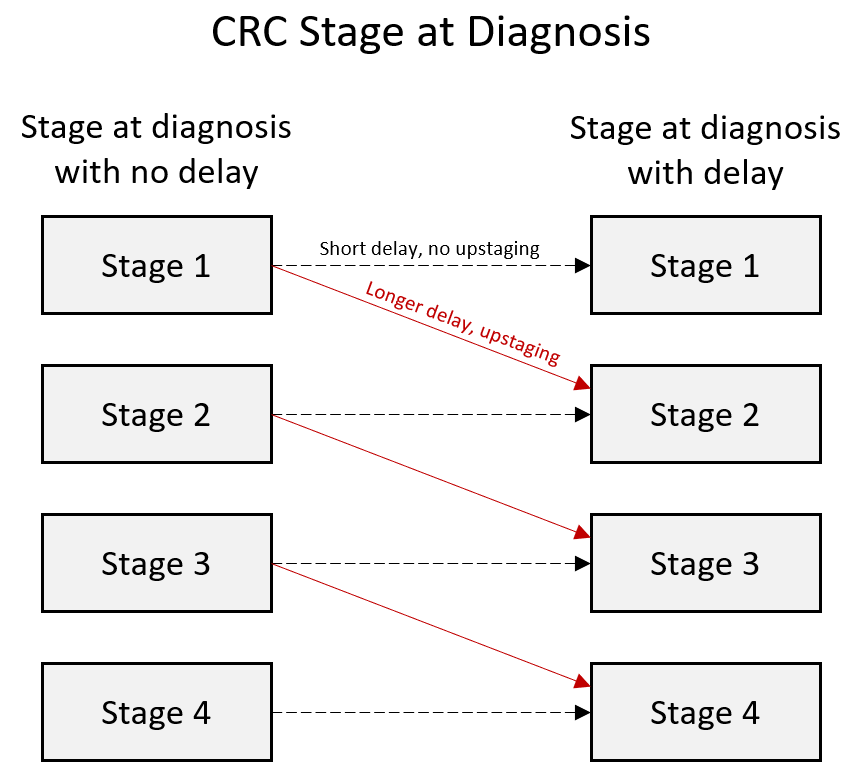

Supplement: S1 Fig — (PNG) [file pone.0296945.s003.png]

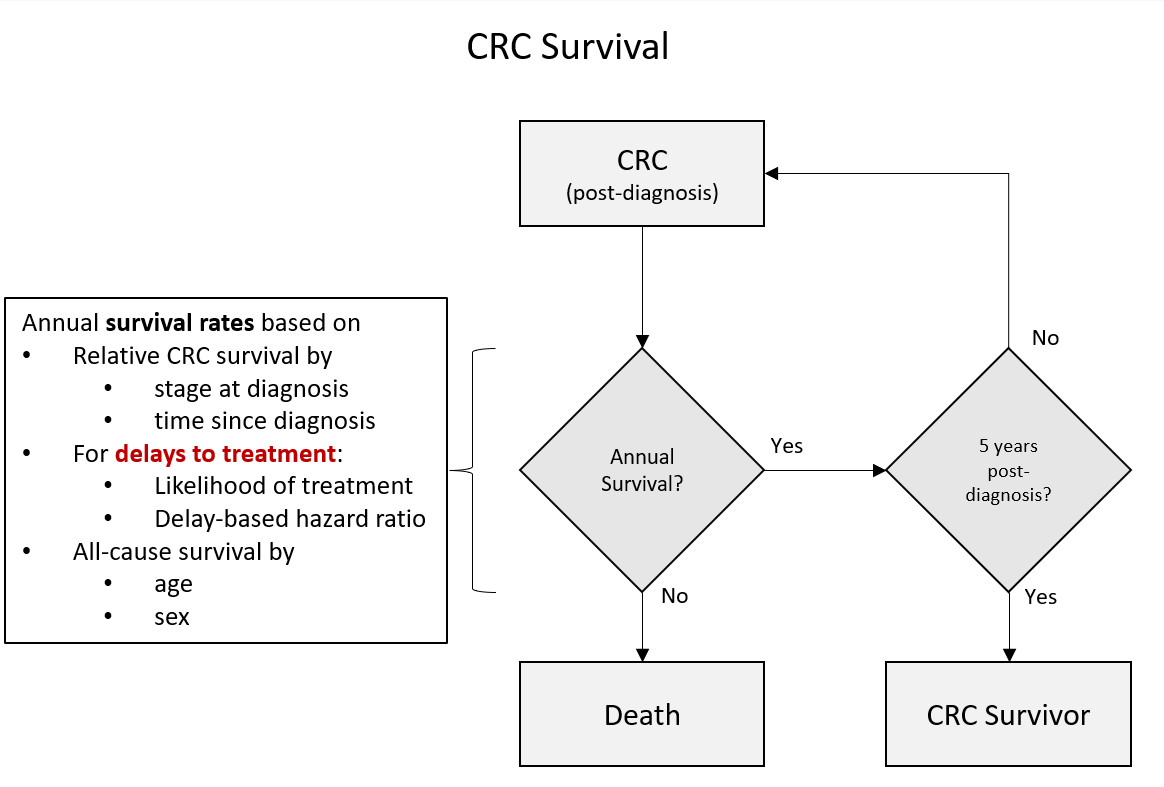

Supplement: S2 Fig — (PNG) [file pone.0296945.s004.png]

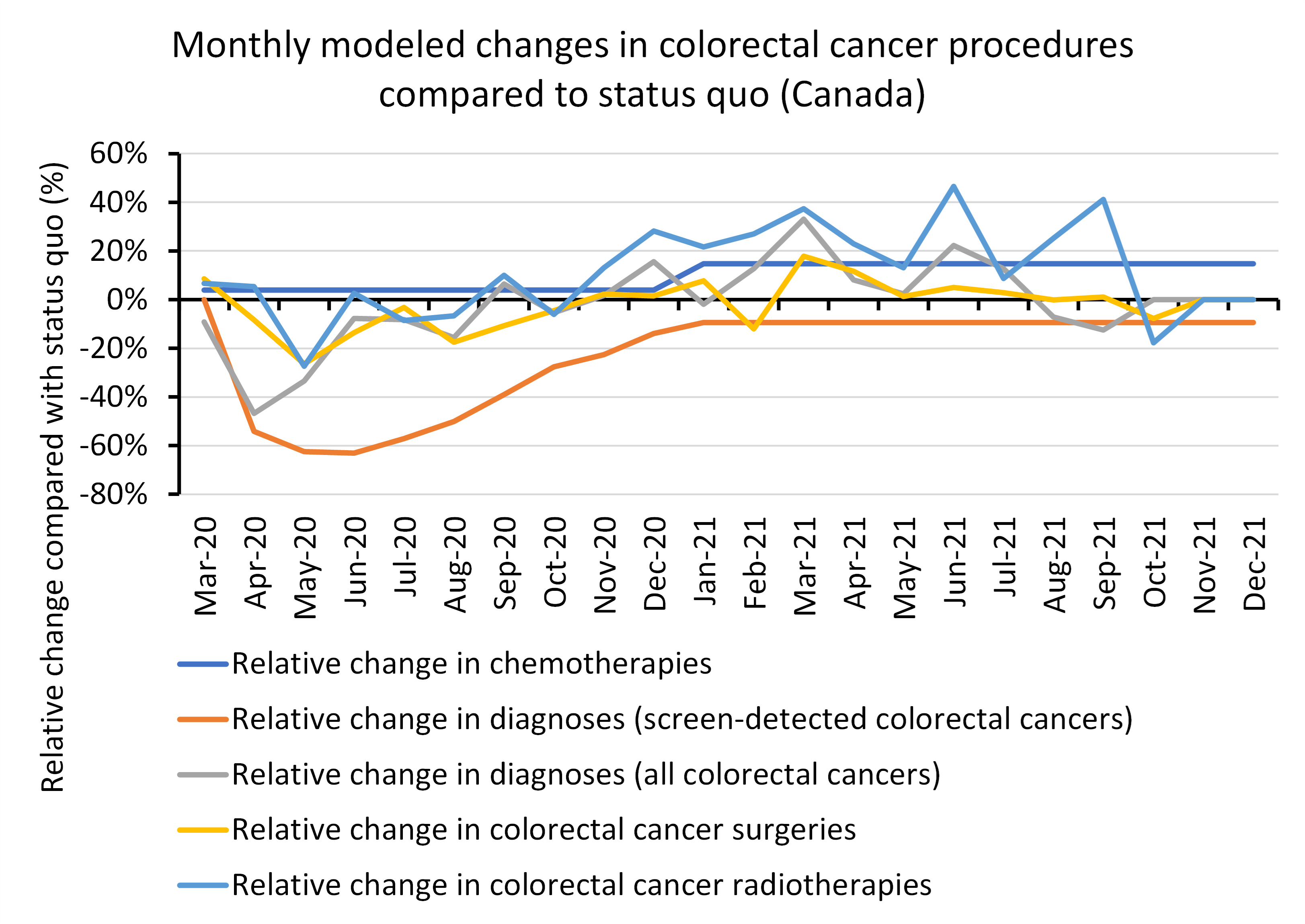

Supplement: S3 Fig — See main text for data sources. (PNG) [file pone.0296945.s005.png]

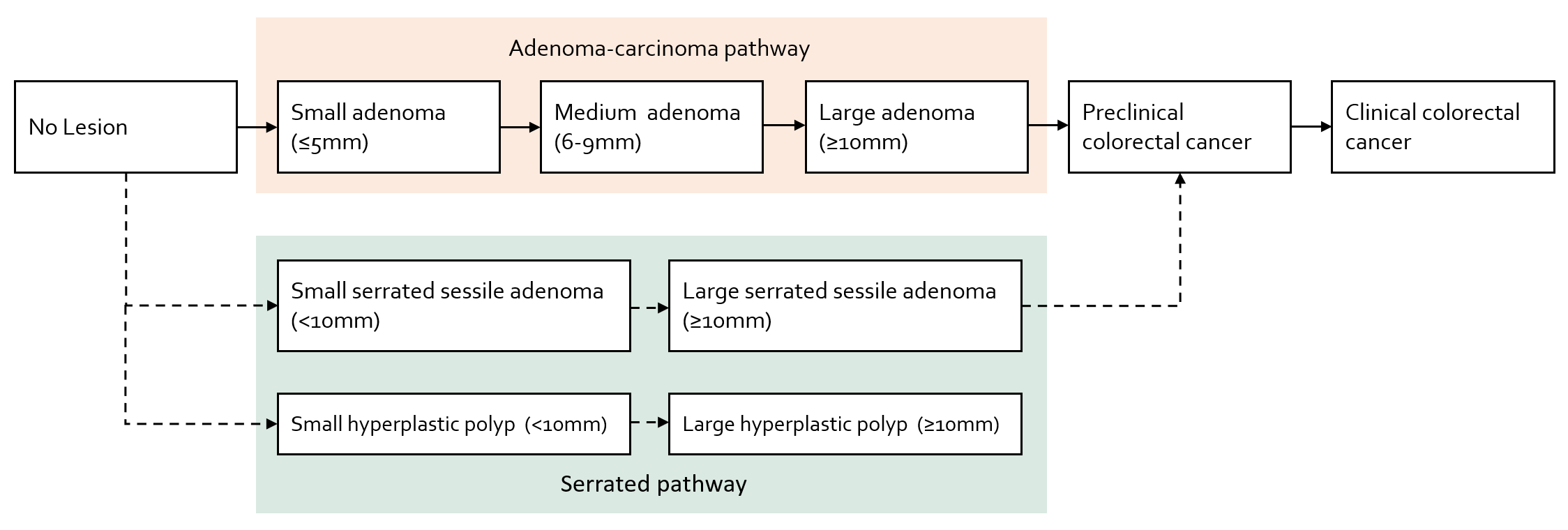

Supplement: S4 Fig — (PNG) [file pone.0296945.s006.png]
